# Protein Phosphatase 4 Promotes Chromosome Pairing and Synapsis, and Contributes to Maintaining Crossover Competence with Increasing Age

**DOI:** 10.1371/journal.pgen.1004638

**Published:** 2014-10-23

**Authors:** Aya Sato-Carlton, Xuan Li, Oliver Crawley, Sarah Testori, Enrique Martinez-Perez, Asako Sugimoto, Peter M. Carlton

**Affiliations:** 1 Institute for Integrated Cell-Material Sciences (iCeMS), Kyoto University, Kyoto, Japan; 2 MRC Clinical Sciences Centre, Imperial College Faculty of Medicine, London, United Kingdom; 3 Laboratory of Developmental Dynamics, Graduate School of Life Sciences, Tohoku University, Sendai, Japan; University of California Santa Cruz, United States of America

## Abstract

Prior to the meiotic divisions, dynamic chromosome reorganizations including pairing, synapsis, and recombination of maternal and paternal chromosome pairs must occur in a highly regulated fashion during meiotic prophase. How chromosomes identify each other's homology and exclusively pair and synapse with their homologous partners, while rejecting illegitimate synapsis with non-homologous chromosomes, remains obscure. In addition, how the levels of recombination initiation and crossover formation are regulated so that sufficient, but not deleterious, levels of DNA breaks are made and processed into crossovers is not understood well. We show that in *Caenorhabditis elegans*, the highly conserved Serine/Threonine protein phosphatase PP4 homolog, PPH-4.1, is required independently to carry out four separate functions involving meiotic chromosome dynamics: (1) synapsis-independent chromosome pairing, (2) restriction of synapsis to homologous chromosomes, (3) programmed DNA double-strand break initiation, and (4) crossover formation. Using quantitative imaging of mutant strains, including super-resolution (3D-SIM) microscopy of chromosomes and the synaptonemal complex, we show that independently-arising defects in each of these processes in the absence of PPH-4.1 activity ultimately lead to meiotic nondisjunction and embryonic lethality. Interestingly, we find that defects in double-strand break initiation and crossover formation, but not pairing or synapsis, become even more severe in the germlines of older mutant animals, indicating an increased dependence on PPH-4.1 with increasing maternal age. Our results demonstrate that PPH-4.1 plays multiple, independent roles in meiotic prophase chromosome dynamics and maintaining meiotic competence in aging germlines. PP4's high degree of conservation suggests it may be a universal regulator of meiotic prophase chromosome dynamics.

## Author Summary

Meiosis creates gametes by distributing diploid genomes containing homologous chromosome pairs into daughter cells that receive only one of each chromosome. To segregate correctly at the first meiotic division, chromosomes must pair and synapse with their homologous partners, and undergo crossover recombination, which requires breaking and repairing the DNA strands of all chromosomes. How chromosomes recognize their partners, and how a cell controls the amount of DNA breakage and recombination that occurs, are open questions. In this study, we observed meiosis in the nematode *Caenorhabditis elegans* to examine the role of Protein Phosphatase 4 (PP4). We found that in the absence of PP4, chromosomes often paired and synapsed with non-homologous chromosomes, or synapsed with themselves by folding in half. Additionally, without PP4 activity, the number of DNA breaks and of crossover recombination events were both independently reduced. The latter two defects became even worse with increasing age, indicating that older animals require PP4 to a greater extent. These findings shed light on how protein phosphorylation controls meiotic events, and demonstrate unanticipated, important roles for PP4.

## Introduction

For a single diploid genome to be partitioned into two haploid genomes in meiosis, chromosomes must undergo a sequence of strictly regulated dynamic events during meiotic prophase. Chromosomes must encounter, assess homology, and form close pairing interactions with their homologous partners, to the exclusion of all other chromosomes. This pairing must then be locked in through synapsis, or the assembly of the synaptonemal complex (SC), which is an intricate protein polymer running the length of each chromosome. Programmed DNA double-strand breaks (DSBs) must also be made by the Spo11 endonuclease to initiate meiotic recombination [1]. A subset of DSBs are repaired as crossovers (COs), exchanges of DNA continuity between maternally- and paternally-derived chromosomes. In most organisms COs are essential for creating links (chiasmata) between homologs that enable their correct segregation into daughter cells. Progression through the series of pairing, synapsis, DSB initiation, and CO formation must be temporally coordinated to coincide with developmental requirements for gamete formation. How chromosomes assess homology, and limit synapsis to homologous partners, is an outstanding mystery. In many organisms, homologous pairing relies on DNA recombination. However, varying levels of homologous alignment can be observed prior to DSB formation in several organisms [2]. In *Caenorhabditis elegans* and *Drosophila melanogaster*, homologous chromosomes pair and synapse in the complete absence of recombination [3], [4]. Since SC proteins containing coiled-coil domains tend to self-assemble [5], SC polymerization must be actively prevented between non-homologous chromosomes. Levels of DSB formation also must be tightly regulated, since creating either too many or too few DSBs is deleterious to the completion of meiosis.

Protein regulation via phosphorylation and dephosphorylation is an essential part of transient responses to cellular events, such as the cell cycle checkpoint or DNA damage response [6]. The reversibility of phosphorylation makes many signaling pathways and feedback regulations possible in a timely manner. In *C. elegans*, kinases such as CHK-2, PLK-1 and -2, and ATM/ATR homologs have been shown to play essential meiotic roles. The inner nuclear envelope protein SUN-1 is phosphorylated in a CHK-2 and PLK-dependent manner when chromosomes begin pairing, and loses its phosphorylation in late pachytene [7]. Failure to finish meiotic tasks such as synapsis or recombination triggers an extension of SUN-1 phosphorylation [8], prolongs the distinct leptotene, zygotene, and early pachytene stages of meiotic prophase [9]–[11], and extends the time window during which DSBs can be made and processed correctly [12], [13]. CHK-2 is predicted to be essential for phosphorylation of other substrates in addition to SUN-1, whereas ATM/ATR kinases regulate many DNA damage repair components in meiosis to ensure correct recombination outcomes [14], [15]. Although the importance of these kinases has been demonstrated, the functions of phosphatases which counterbalance these kinases during meiotic prophase have received comparatively little attention, and remain ill-understood.

In *C. elegans*, RNAi depletion of the *pph-4.1* gene (encoding a homolog of the catalytic subunit of the PP4 holoenzyme) has previously been shown to result in the appearance of more than the diploid number of 6 chromosome pairs in late meiotic prophase, indicating a failure to form chiasmata [16]. Since any errors in chromosome pairing, synapsis, or recombination could result in failure to create chiasmata, which processes PPH-4.1 directly regulates during meiotic prophase remains an open question. It has been shown that budding yeast PP4 controls the non-homologous clustering of centromeres in early meiotic prophase through dephosphorylation of Zip1, an SC central element protein. Additionally, PP4 is independently required for complete SC formation in budding yeast. [17]. Nonhomologous centromere pairing is thought to improve segregation of nonexchange chromosomes by holding them together until anaphase I [18], [19]. This non-homologous coupling of centromeres at the onset of meiosis has been observed in yeast and some plants [20], [21], but its absence from animal meiosis suggests that the meiotic functional repertoire of PP4 has yet to be elucidated.

In this work, we have discovered that four essential steps in meiotic prophase require PPH-4.1 activity: (1) synapsis-independent chromosome pairing, (2) prevention of nonhomologous synapsis, (3) programmed DSB initiation, and (4) post-DSB CO formation. The combined failure of all these processes in cells lacking PPH-4.1 activity leads ultimately to significant numbers of chromosomes without chiasmata, chromosome nondisjunction, and embryonic lethality. In contrast to yeast PP4 mutants that are defective in SC assembly, we find that *C. elegans pph-4.1* mutants have robust but premature SC assembly between nonhomologous chromosomes or on folded-over single chromosomes. We further demonstrate that DSB initiation and CO formation, but not chromosome pairing, increase their dependence on PPH-4.1 in an age-dependent manner, suggesting an increased requirement for PPH-4.1 to make sufficient numbers of DSBs and COs in older animals. Since PPH-4.1 in *C. elegans* is 92% identical at the amino acid level with human PP4C, it is likely that the roles we have discovered for PPH-4.1 have functionally conserved parallels in human meiosis.

## Results

### Loss of PPH-4.1 phosphatase activity results in meiotic defects that worsen with age

**Figure 1:**
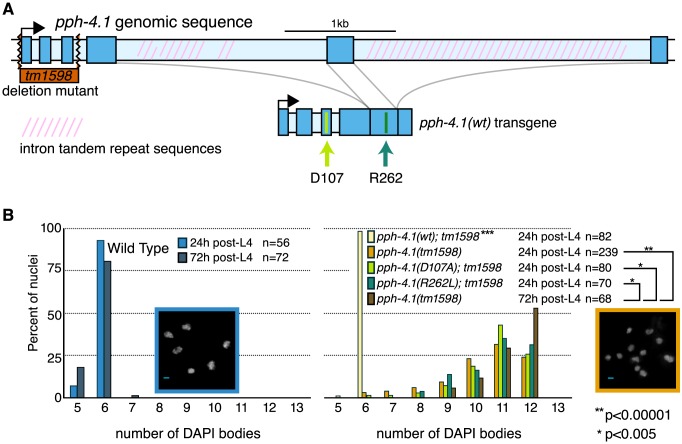
Mutations in the *pph-4.1* gene lead to loss of chiasmata. (A) Schematics of the *pph-4.1* gene, deletion allele, and transgenes constructed in this study. (B) Age-dependent failure to create chiasmata at meiosis. The number of DAPI-staining bodies are shown as percentages of the indicated number of late prophase oocytes scored for each genotype. Image insets show a wild-type nucleus (left) and a *tm1598* mutant nucleus (right) at 24 h post-L4.

We characterized the predicted null allele *pph-4.1(tm1598)*, which deletes the first three exons of the *pph-4.1* coding sequence (Figure 1A). No evidence of maternal protein carryover was detected in *pph-4.1* homozygous adults (Figure S1). Examination of self-progeny of mutant hermaphrodites showed that *tm1598* has low embryo viability (3%) with a high incidence of males (23.8%), indicative of X chromosome nondisjunction, in the surviving progeny (Table S1). Cross-progeny of mutant hermaphrodites with wild-type males showed significantly higher embryonic viability (9.8%), indicating both spermatogenesis and oogenesis are affected in *pph-4.1* hermaphrodites. To characterize meiotic defects, we dissected gonads going through oogenesis from *pph-4.1* hermaphrodites and scored the number of DAPI-stained chromosomes (DAPI bodies). In a wild-type hermaphrodite, the six pairs of *C. elegans* chromosomes give rise to six chiasmate bivalents at diakinesis (late meiotic prophase), demonstrating the successful formation of crossovers between all six pairs of homologous chromosomes. On the other hand, the presence of 7 or more DAPI-staining bodies in diakinesis oocytes indicates the failure of one or more chromosome pairs to undergo crossover formation. Similarly to previously-shown RNAi depletion [16], *pph-4.1(tm1598)* mutant homozygotes showed frequent univalent formation (Figure 1B).

Interestingly, the univalent phenotype of *pph-4.1* mutants grew worse with age: in adult worms 72 hours after the L4 larval stage (72 h post-L4), the distribution of univalents significantly shifts toward higher numbers, such that over 50% of diakinesis nuclei contained 12 DAPI bodies, indicating that none of the six chromosome pairs in those nuclei succeeded in chiasma formation. This rate of failure was seen in less than 25% of nuclei in 24 h post-L4 mutant worms. The average number of bivalents per *pph-4.1* nucleus was 1.55 at 24 hours post-L4, and 0.71 at 72 hours post-L4, indicating that roughly half of the already-compromised meiotic competence is lost over 48 hours in *pph-4.1* mutants.

To determine whether PPH-4.1 phosphatase activity is specifically required for meiosis, we constructed two phosphatase-dead transgenes containing single amino acid substitutions (D107A or R262L), analogous to known mutations in the active site of mammalian PP4C that lead to loss of catalytic activity [22], [23], and created transgenic *C. elegans* lines with each construct. Although both mutant proteins were expressed at levels similar to wild-type PPH-4.1 (Figure S1A), both *pph-4.1(D107A)* and *pph-4.1(R262L)* mutant gonads showed defective bivalent formation, similar to the null *tm1598* allele. As a control, we constructed a single-copy transgene *pph-4.1(WT)* with wild-type *pph-4.1* coding sequence, but otherwise identical in structure and MosSCI insertion site to the point mutants. *pph-4.1(tm1598)* mutant worms homozygous for *pph-4.1(WT)* had 6 bivalents per nucleus, indicating a full rescue of the mutation (Figure 1B). Since the inability of either mutant transgene to rescue the *pph-4.1* phenotype can be solely attributed to the point mutations, we conclude that the phenotype of the *pph-4.1(tm1598)* allele is also specifically due to the loss of phosphatase activity.

### PPH-4.1 is required for autosomal pairing

**Figure 2:**
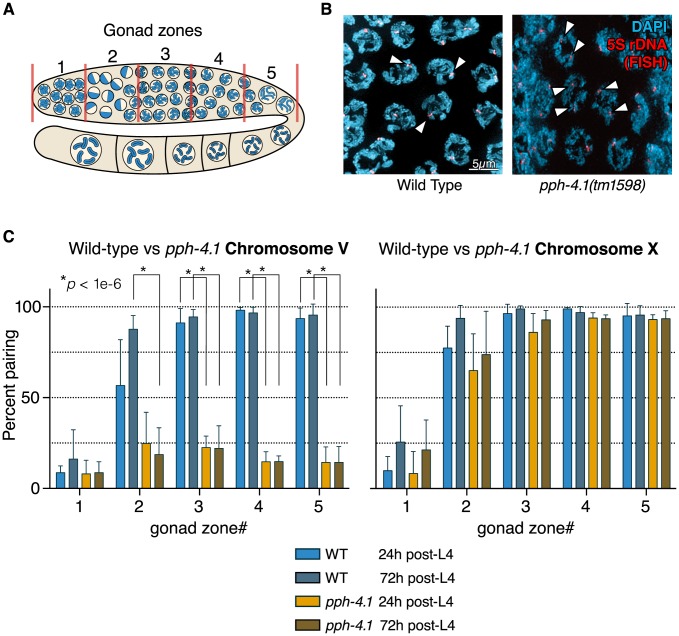
Autosomal pairing is diminished in *pph-4.1* mutants. (A) Schematic showing hermaphrodite gonads divided into 5 equally-sized zones for scoring. (B) FISH images demonstrate paired 5S rDNA sites in wild-type (left; arrowheads indicate paired foci) and unpaired sites in *pph-4.1* mutants (right; arrowheads indicate unpaired foci) at pachytene. (C) quantitation of pairing for chromosome V (left) and X (right) shown as the percent of nuclei with paired signals in each zone. Error bars show standard deviation. Six gonads were scored for each genotype. The total number of nuclei scored for zone 1,2,3,4,5 respectively was as follows: WT 24 h pL4: 293, 337, 409, 410, 222; wt 72 h pL4: 283, 322, 333, 314, 184; *pph-4.1* mutant 24 h pL4: 237, 333, 303, 297, 269; *pph-4.1* mutant 72 h pL4: 318, 340, 393, 347, 305.

To further characterize the meiotic defects of the *tm1598* allele, we next analyzed homologous chromosome pairing in young (24 h post-L4) and old (72 h post-L4) animals. We monitored pairing of one locus on the right arm of chromosome V with fluorescence *in situ* hybridization (FISH) probes that label the 5S rDNA locus. Pairing of the X chromosome was visualized by immunofluorescence against HIM-8, which binds to the left end of the X chromosome at the *cis*-acting pairing center (PC) [11]. Taking advantage of the fact that the distal-to-proximal position of meiocytes in the *C. elegans* gonad mirrors the temporal progression of meiotic prophase, we scored pairing over time by measuring the fraction of nuclei containing paired versus unpaired signals within each of 5 equal-length zones of the distal gonad (Figure 2A,B). In contrast to wild-type animals which displayed increasing pairing of chromosome V, eventually reaching 100%, *pph-4.1* animals never achieved pairing levels higher than 30% (Figure 2C). In marked contrast to chromosome V, the X chromosome PC in *pph-4.1* animals was found to achieve pairing frequencies indistinguishable from wild-type, and with the same kinetics (Figure 2C). We found similar discrepancies between X and autosome pairing behavior when examining the right end of the X with FISH, and chromosomes I and IV with immunostaining of ZIM-3, a protein localizing to pairing center ends of chromosomes I and IV [24] (Figure S2A, B). As expected from their higher pairing frequency, X chromosomes are significantly more likely to remain bivalent at diakinesis than chromosome V (Figure S2C). No difference in pairing ability was observed between 24 h post-L4 and 72 h post-L4 for either the X chromosome or chromosome V in *pph-4.1* animals. Therefore, the age-related loss of chiasma formation in *pph-4.1* mutants is not attributable to decreased chromosome pairing.

### SC assembles completely, but synapsis-independent pairing is defective in *pph-4.1* mutants

**Figure 3:**
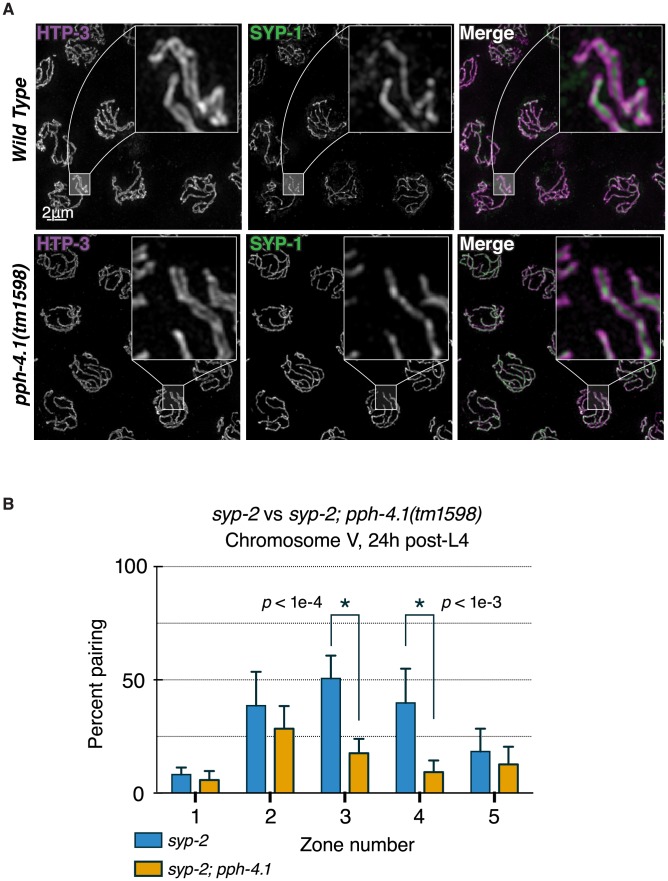
Canonical SC structure but reduced synapsis-independent pairing in *pph-4.1* mutants. (A) 3D-SIM images of pachytene nuclei immunostained for axial element HTP-3 (violet in merged image) and central element SYP-1 (green). Boxed insets at 5x higher magnification demonstrate position of SYP-1 between parallel tracks of HTP-3. (B) quantitation of SC-independent pairing of 5S rDNA loci in *syp-2* and *syp-2; pph-4.1* mutants. The percent of nuclei with paired foci in each of 5 zones (see Figure 2) is shown; error bars show SD. Six gonads were scored for each genotype. The total number of nuclei scored for zones 1–5 was as follows: *syp-2* single mutant: 294, 322, 427, 417, 249; *syp-2; pph-4.1* double mutant: 268, 281, 245, 295, 251.

Since *pph-4.1* mutants exhibited reduced autosomal pairing, we next decided to assess the nature and extent of SC formation, through immunostaining of SC proteins. The SC is a zipper-like structure consisting of two lateral elements that run the length of each chromosome, and a central element that bridges the 100 to 200 nm distance between the lateral elements [25]. Since this distance is smaller than the diffraction limit of visible light, we visualized SCs of *pph-4.1* mutant animals with three-dimensional structured illumination microscopy (3D-SIM) [26], which permits the resolution of SC lateral elements as two separate strands with central elements located in the middle [27]. By visualizing the central element protein SYP-1 and the lateral element protein HTP-3, we found that *C. elegans pph-4.1* mutants nearly always formed complete SC, with SYP-1 localizing between parallel HTP-3 axes separated by approximately 150 nm (Figure 3A). This observation supports the conclusion that SC structure in *pph-4.1* mutants is canonical and *pph-4.1* activity is not required to form the SC itself. This result contrasts with observations from budding yeast, in which SC formation itself depends on PP4 [17].

Since *pph-4.1* mutants displayed normal SC structure but mispairing of allelic loci, we concluded that SC must be forming between non-homologous chromosomes. This led us to consider whether non-homologous synapsis could be responsible for autosomal mispairing in *pph-4.1* mutants, by trapping chromosomes with the wrong partner. To assess this possibility, we examined autosomal pairing in worms lacking the SC central element protein SYP-2. Meiotic prophase chromosomes in *syp-2* mutant animals do not form SC, but engage in appreciable levels of synapsis-independent homologous pairing [10]. We observed levels of homologous pairing in *syp-2* single mutants (Figure 3B) that were in agreement with previous studies. In contrast, *syp-2; pph-4.1* double mutants displayed significantly lower pairing levels, indistinguishable from *pph-4.1* single mutants. Since the defect in chromosome V pairing in *syp-2; pph-4.1* mutants cannot be explained by promiscuous SC formation, we conclude that PPH-4.1 activity is required for the synapsis-independent pairing of autosomes.

### Characterization of nonhomologous synapsis in *pph-4.1* mutants with 3D-SIM

**Figure 4:**
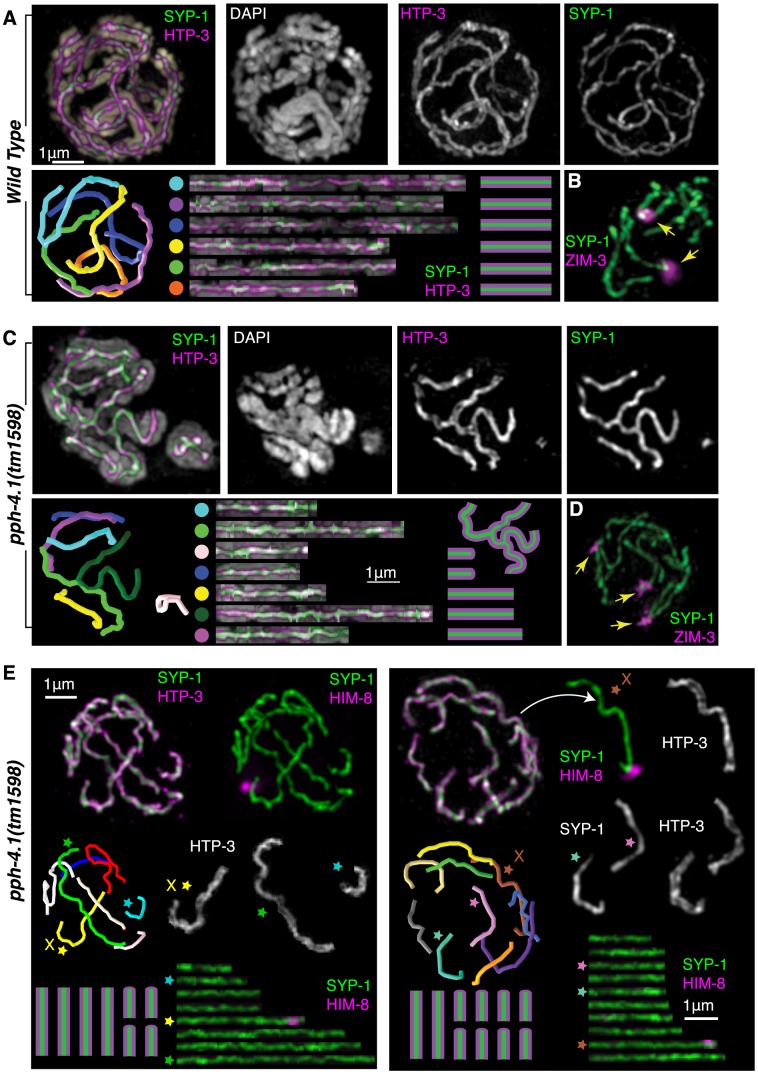
Multiple synaptic aberrations are found in *pph-4.1* mutants. (A) 3D-SIM image of synapsed chromosomes in a wild-type nucleus. Top row shows maximum-intensity projections of image data in multiple channels; bottom row shows computer-aided traces of the six paired chromosomes. Correspondences between computer model (left) and straightened chromosomes (right) shown by colored dots. (B) A wild-type nucleus stained for SYP-1 and ZIM-3 showing two ZIM-3 foci at the synapsed PC ends of chromosomes I and IV. (C) 3D-SIM image of a *pph-4.1* nucleus shown in maximum-intensity projection of the entire nucleus (leftmost image, color) and a subset of Z sections (individual grayscale channels) highlighting a nonhomologously synapsed quartet of chromosomes, each making one or two switches of pairing partner. Computer traces (left) show seven individual strands, indicating two chromosomes likely undergoing foldback synapsis in the same nucleus. (D) *pph-4.1* nucleus stained for SYP-1 and ZIM-3 shows three synapsed foci, indicating non-homologous synapsis. (E) Highlighted examples of aberrant synapsis in two *pph-4.1* nuclei. HTP-3, SYP-1, and HIM-8 are shown to highlight axial elements, central elements, and the X chromosome. Straightened chromosome images are starred to correspond to individual chromosomes in the 3D traces. All chromosome configurations shown in schematic are inferred from straightened chromosome lengths and the requirement that 12 individual chromosomes are involved.

To quantitatively confirm the nature of the nonhomologous synapsis we inferred, we traced the three-dimensional paths of wild-type and *pph-4.1* SCs in 3D-SIM images. Wild-type nuclei at late pachytene invariably showed full-length synapsis of all 6 chromosome pairs (Figure 4A). In contrast, we observed a variety of synaptic aberrations in many *pph-4.1* nuclei, including full-length synapsis of nonhomologous chromosomes, multivalent synapsis between three or more chromosomes and self-synapsis of unpaired chromosomes, which we infer to be foldback synapsis based on length (Figure 4C,E). Manual tracing of pachytene chromosome complements from wild-type and *pph-4.1* nuclei showed that 20 out of 20 wild-type nuclei had six fully-synapsed chromosomes, whereas 15 out of 20 *pph-4.1* nuclei had synaptic aberrations detectable by 3D-SIM imaging of SYP-1 and HTP-3 staining (Figure S3). Staining of the ZIM-3 protein, which binds to the PCs of chromosomes I and IV, often revealed more than two synapsed foci in *pph-4.1*, but not in wild-type nuclei (Figure 4B, D), indicating full-length synapsis of distinct non-homologous chromosomes. In contrast to the autosomal PCs, the X chromosome PC was nearly always both paired and synapsed homologously in *pph-4.1* mutants (Movie S1). Homologous synapsis of the X chromosome, but not the autosomes, is also a consequence of mutations in the axial element gene *htp-1 or him-3* [28]–[30]; we therefore performed immunostaining to examine whether HTP-1/2 and HIM-3 proteins are normally localized to the SC in *pph-4.1* mutants. We observed robust loading of HTP-1/2 and HIM-3 onto axes concomitant with HTP-3 in *pph-4.1* mutants (Figure S4); therefore, the nonhomologous synapsis phenotype cannot be explained by a failure of HTP-1/2 or HIM-3 to load onto chromosomes.

### PPH-4.1 is required for wild-type levels of DSB initiation

The extent of nonhomologous pairing and synapsis we observed did not fully explain the high frequency of univalent chromosomes at diakinesis. Although the X chromosomes pair and synapse at nearly 100% frequency in *pph-4.1* animals, they must nevertheless fail to form chiasmata in at least 25% and 50% of cases in young and old adults, respectively, based on our observed frequencies of nuclei containing 12 univalents. Since failure to form chiasmata despite successful pairing suggests problems with recombination, we next assessed recombination in wild-type and *pph-4.1* mutant animals.

**Figure 5:**
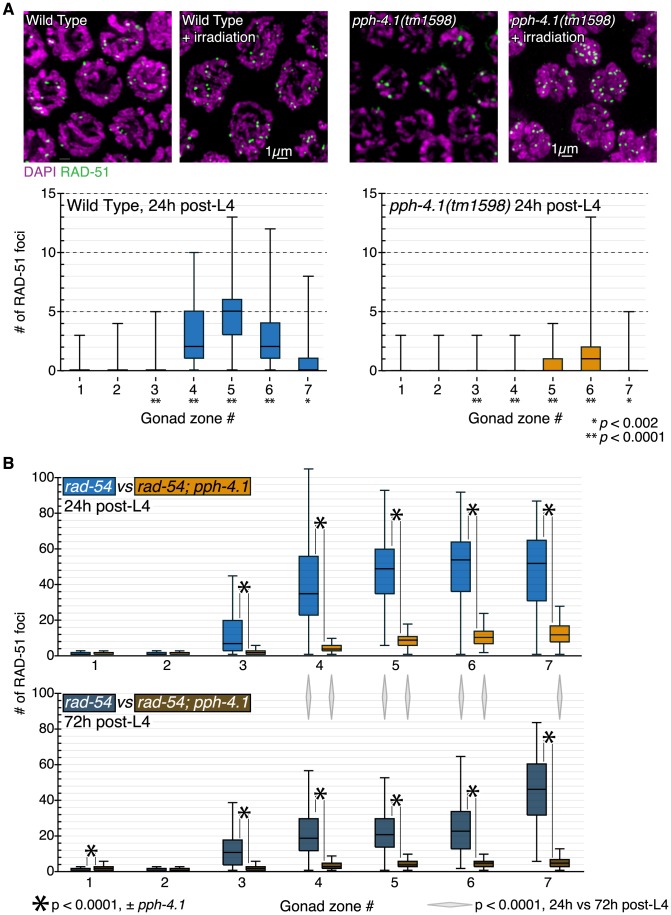
DSB initiation is perturbed in an age-dependent manner in *pph-4.1* mutants. (A) Wild-type and *pph-4.1* nuclei shown with DAPI staining in magenta and α-RAD-51 staining in green. *Top*, γ-irradiation at 10Gy restores RAD-51 staining to *pph-4.1* nuclei. *Bottom*, quantitation of RAD-51 focus formation in wild-type and mutant animals. RAD-51 focus numbers are depicted as a box plot, with box indicating mean and quartiles. Significance was assessed via the Mann-Whitney test. Three gonads were scored for each condition; the numbers of nuclei scored in zones 1–7 are as follows: for wild-type, 316, 312, 256, 252, 231, 198, 120; for *pph-4.1*, 231, 244, 237, 245, 231, 205, 136. (B) Quantitation of RAD-51 foci with increasing maternal age. Numbers of foci in each of 7 zones are depicted with box plots as in (A). *Top*, focus numbers compared between *rad-54* and *rad-54; pph-4.1* animals at 24 h post-L4. *Bottom*, comparison at 72 h post-L4. Asterisks indicate significant differences due to loss of *pph-4.1*; diamonds between top and bottom graphs show significance due to age; comparisons were performed via the Mann-Whitney test. Three gonads were scored for each condition; the numbers of nuclei scored in zones 1–7 are as follows: for *rad-54* 24 h, 252, 313, 443, 397, 311, 236, 111; for *rad-54* 72 h, 237, 321, 397, 467, 395, 268, 64; for *rad-54; pph-4.1* 24 h, 288, 288, 306, 359, 300, 232, 70; for *rad-54; pph-4.1* 72 h, 255, 230, 262, 251, 229, 218, 118.

First, we performed immunostaining against the strand-exchange protein RAD-51 in wild-type and *pph-4.1* mutants, and quantified RAD-51 focus number per nucleus in each of seven equal-length zones of the distal gonad. RAD-51 foci became visible in wild-type gonads after the transition zone, and their number peaked in mid-pachytene with an average of around 5 foci per nucleus (Figure 5A). Most *C. elegans* mutants with unpaired or incorrectly paired chromosomes accumulate RAD-51 numbers that exceed wild-type levels, due to the inability to repair recombination intermediates from a homologous chromosome template [10], [31], [32]. However, *pph-4.1* gonads displayed greatly reduced RAD-51 focus numbers. We also observed reduced levels of the single-strand binding protein RPA-1 in the mutant compared to the control (Figure S5). These findings raised the possibility that RAD-51 and RPA-1 loading at DSB sites requires PPH-4.1 activity. To test this, we induced DSBs by exposing worms to 10Gy of γ-rays at 24 h post-L4, and visualized early pachytene RAD-51 foci 2 hours later. We found that RAD-51 focus numbers had increased in irradiated mutant animals, to a level qualitatively comparable to irradiated wild-type animals (Figure 5A), suggesting that *pph-4.1* mutants have a reduced number of programmed DSBs, but are competent to load recombination proteins onto any breaks that exist.

Next, we assessed whether lowered RAD-51 levels in *pph-4.1* mutants could be explained by fast, premature repair. In meiotic prophase, programmed DSBs are preferentially repaired using the homologous chromosome as a template, rather than the sister chromatid [33]. However, when interhomolog bias is defective, DSBs can be rapidly repaired from the sister chromatid [29]. To test if this were the case in *pph-4.1* mutants, we examined RAD-51 focus formation in the *rad-54* mutant background, in which DSBs are created but cannot be repaired, leaving RAD-51 foci to persist [34], [35]. In *rad-54* mutants, the number of RAD-51 foci thus reflects the total number of DSBs created. In accordance with previous observations [36], *rad-54* single mutants displayed RAD-51 focus numbers much higher than wild-type (Figure 5B, Figure S6). In contrast, *rad-54; pph-4.1* double mutants had significantly fewer RAD-51 foci compared to *rad-54* single mutants, indicating the net number of DSB initiations is reduced in *pph-4.1* mutants. These results lead us to conclude that loss of PPH-4.1 specifically compromises the ability to make wild-type levels of programmed DSBs. *rad-54; pph-4.1* double mutants had significantly more RAD-51 foci than *pph-4.1* single mutants, demonstrating that nuclei without PPH-4.1 are still capable of making appreciable numbers of DSBs. However, this residual DSB initiation activity in the absence of PPH-4.1 decreases with maternal age: in *rad-54; pph-4.1* animals at 72 h post-L4, the number of RAD-51 foci attains a level that is roughly half that seen at 24 h post-L4 in all zones after RAD-51 foci first form. Interestingly, the number of RAD-51 foci in *rad-54* single mutants is also significantly lower at 72 h post-L4, compared to 24 h post-L4, in zones 4, 5, and 6, suggesting that reduction of DSB initiation may be intrinsic to aging.

### PPH-4.1 is required for wild-type levels of COs

**Figure 6:**
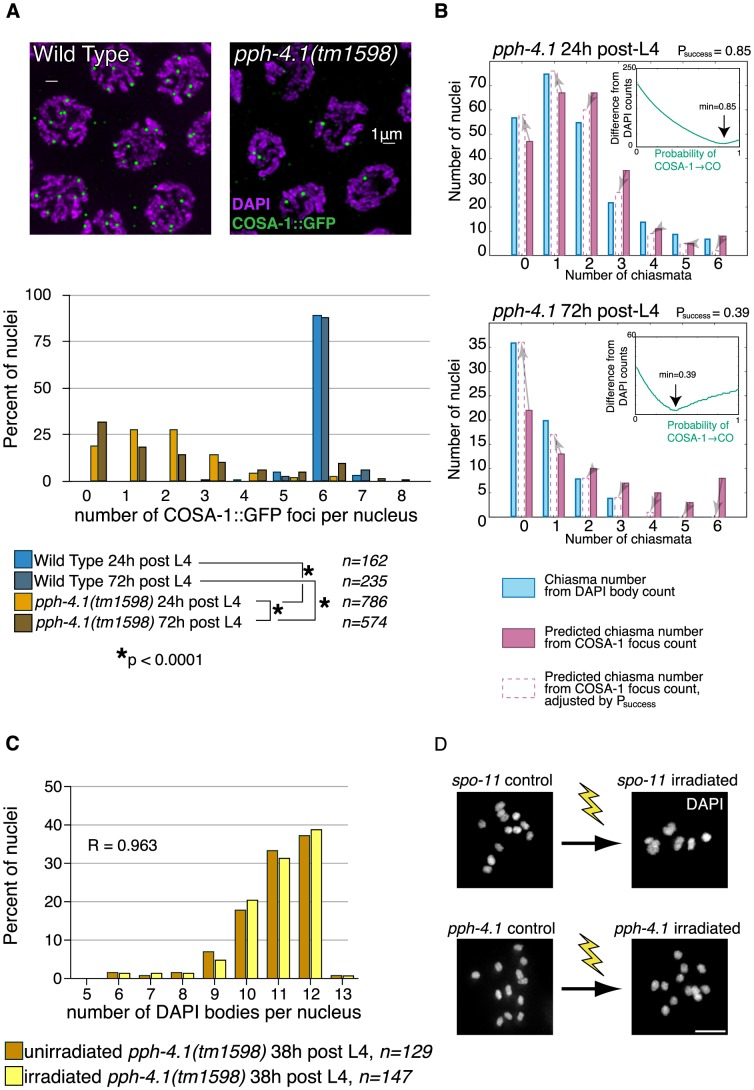
COSA-1 foci are reduced, and γ-irradiation does not rescue bivalent formation in *pph-4.1* mutants. (A) COSA-1 staining suggests reduced COs in the *pph-4.1* mutant. *Top*, meiotic cells at pachytene in WT (left) and *pph-4.1* (right). *Bottom*, quantitation of COSA-1 focus number. *p* value of chi-square test is shown. Scale bar, 2 µm. (B) Estimation of age-dependent probabilities of COSA-1 sites to successfully mature into COs. Inset graphs show the sum of the squared differences between the DAPI body-inferred and the COSA-1 focus-inferred chiasma numbers as a function of varying P_success_. The minimum value (used to generate the bar graph adjustments) is indicated with an arrow. (C) A frequency histogram (normalized to 100%) shows DAPI body numbers for *pph-4.1* control (blue bars) and irradiated (green bars) nuclei. R, Spearman's rank correlation coefficient. (D) γ-irradiation restored chiasmata on all 6 chromosome pairs in *spo-11(me44)* gonads (top) but did not produce additional chiasmata in *pph-4.1* gonads (bottom). Scale bar, 5 µm.

We next inquired whether PPH-4.1 was required for recombination at steps subsequent to DSB formation. First, we assessed the number of presumptive CO sites in wild-type and *pph-4.1* mutant animals by detection of COSA-1 (Figure 6A), a protein shown to localize to sites designated for CO repair in *C. elegans* [37]. COs in many organisms are subjected to the phenomenon of interference, in which CO formation inhibits the formation of further COs nearby. In *C. elegans*, this interference operates over the length of entire chromosomes, limiting COs to one per chromosome pair [38], [39], resulting in 6 COSA-1 foci in wild-type meiotic pachytene nuclei [37]. We started to detect COSA-1:GFP foci in mid-pachytene and observed nearly 100% occurrence of 6 COSA-1 foci per nucleus in late pachytene, 1 per chromosome pair, in control animals. The number of COSA-1 foci in each late pachytene nucleus was 6 in both 24 h and 72 h post-L4 control animals. In contrast, in *pph-4.1* mutants, we observed a significant reduction in COSA-1 foci, with a significant proportion of nuclei having no foci. Additionally, the number of COSA-1 foci in *pph-4.1* underwent an even further decrease with advancing maternal age: in mutant animals at 72 h post-L4, the distribution of focus numbers shifted significantly towards zero compared to 24 h post-L4 animals, suggesting the creation of fewer COs. These observations qualitatively agree with the increasing number of DAPI bodies observed in older animals. However, using COSA-1 focus numbers to predict the observed number of DAPI bodies from the same time points in Figure 1 reveals a positive offset (Figure 6B): the number of COSA-1 foci exceeds the predicted number of chiasmata in both 24 h and 72 h post-L4 animals. This discrepancy can be accommodated by postulating probabilities less than 100% for COSA-1 foci to mature into a CO in *pph-4.1* mutants; adjusting for lower probabilities gave predicted chiasma distributions that more closely match the observed DAPI body numbers. For 24 h post-L4 worms, a success rate of 85% led to an optimal match between DAPI body numbers and COSA-1 foci, while for 72 h post-L4 worms the optimally-matching rate was 39%. The decrease in the correlation between COSA-1 foci and chiasmata suggests that in the *pph-4.1* mutant, advancing age leads to fewer COs in two ways: by reducing the initial number of COSA-1 foci, and also reducing the probability of a COSA-1 focus maturing into a chiasma.

To examine further whether CO formation capacity requires PPH-4.1 as inferred from the COSA-1 data, we took advantage of the fact that the X chromosome is normally paired and synapsed in *pph-4.1* mutants (Movie S1). If the dearth of chiasmata on the X chromosome were solely attributable to reduced DSB formation, then irradiation-induced DSBs ought to allow the X chromosomes to receive a chiasma in many cases, since chiasma failure caused by a lack of DSBs can be rescued by inducing artificial breaks with γ-rays [3]. Similar considerations for the autosomes, which attain low but non-negligible levels of homologous synapsis, suggested that increasing DSB number through irradiation should result in a measurable shift toward fewer univalent chromosomes (and thus fewer observed DAPI bodies) at diakinesis. Contrarily, if PPH-4.1 were required for carrying out post-DSB steps of CO formation at a wild-type level of competence, then creating new DSBs would not necessarily lead to a reduction in unpaired chromosomes. To test these possibilities, we exposed *pph-4.1* animals at 20 h post-L4 to 10 Gy of γ-rays to induce DSBs, and counted DAPI bodies in diakinesis nuclei 18 hours later. We found no difference in the distribution of univalents between irradiated and non-irradiated *pph-4.1* mutants (Figure 6C). We confirmed the ability of the given dose of γ-rays to cause DSBs by irradiating *spo-11(me44)* animals in parallel, and observing a significant increase in bivalent numbers, compared to unirradiated controls (Figure 6D). Since the artificial introduction of DSBs in the *pph-4.1* mutant did not lead to a detectable decrease in univalent number, in spite of the abundance of homologously synapsed X chromosomes, we conclude that PPH-4.1 is required for wild-type levels of CO formation in addition to its roles in pairing, synapsis, and DSB initiation.

Since a previous study showed that PP4 promotes crossover interference in budding yeast [17], we decided to test whether the normal operation of interference was intact in *pph-4.1* mutants. We irradiated worms 18 h post-L4 with 10 Gy of γ-rays, and examined COSA-1 foci 8 h post-irradiation. We found 1 out of 227 control nuclei, and 3 out of 189 *pph-4.1* mutant nuclei, displaying two COSA-1 foci on a single HTP-3 stretch. Since this difference is not significant (P = 0.3338, Fisher's exact test), we conclude that the mechanism limiting COSA-1 foci to one per chromosome in *C. elegans* does not require PPH-4.1 for its function.

### Altered meiotic progression and SUN-1 phosphorylation in *pph-4.1* mutants

**Figure 7:**
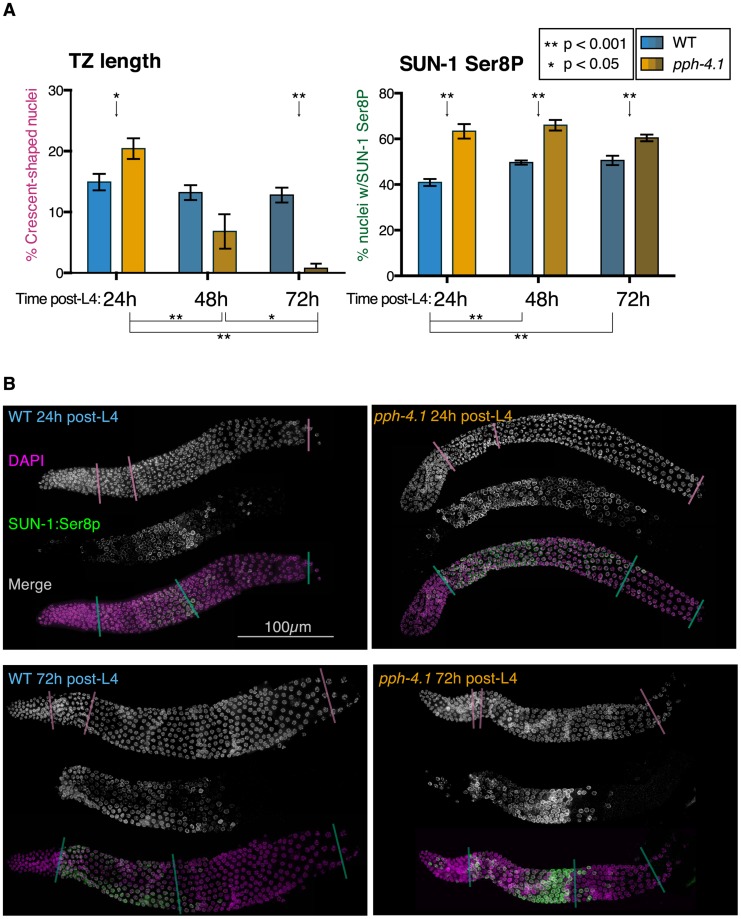
Transition zone nucleus numbers are abnormal, and SUN-1 phosphorylation is extended, in *pph-4.1* mutants. (A) Comparison of transition zone length (left) and SUN-1:Ser8p length (right) in wild-type and *pph-4.1* mutant worms at 24 h, 48 h, and 72 h post-L4. Percentage of gonad length showing transition zone nucleus morphology based on DAPI staining (left) or showing SUN-1:Ser8p staining (right) is normalized to the gonad length from the transition zone until cellularization. Error bars show SEM; *n* = 8 gonads scored for all conditions. (B) Representative gonad images of wild-type and *pph-4.1* mutant worms are shown at 24 h and 72 h post-L4. A single gonad arm is shown in each subpanel: grayscale images show DAPI staining of nuclei (magenta in merged image) and SUN-1:Ser8p immunostaining (green); color images show the channels merged in magenta and green. Color-coded lines through gonads demarcate the beginning and end of the transition zone (scored by DAPI morphology) or of SUN-1:Ser8p staining, and the beginning of oocyte cellularization, as used for quantitation in Figure 7A.

Many meiotic mutations causing non-homologous synapsis result in a shorter region of the leptotene/zygotene transition zone marked by crescent-shaped nuclei with unresolvable chromosomes, as well as promiscuous loading of SC central elements [28], [29], [32]. In contrast, we observed that *pph-4.1* animals at 24 h post-L4 had longer transition zone regions as scored by nuclear morphology, compared to the wild-type (Figure 7). However, transition zone lengths dramatically and unexpectedly decreased with age in *pph-4.1* mutants. In 72 h post-L4 *pph-4.1* mutants, seven out of eight gonads measured had very few leptotene/zygotene nuclei. In these gonads, nuclei progressed directly from a premeiotic appearance to an early pachytene appearance. This transition is accompanied by immediate loading of the central element of the SC (Figure S7A) after the mitotic zone, suggesting that as *pph-4.1* mutants age, synapsis cannot be delayed in response to the lack of homologous pairing. At 48 h post-L4, transition zone lengths in *pph-4.1* animals were highly variable and overlapped both the 72 h and 24 h distributions, suggesting that loss of transition zone morphology occurs at around 48 h post-L4 in *pph-4.1* mutants. The age-dependent loss of transition zone nuclei and earlier appearance of full-length synapsis in *pph-4.1* mutants suggests that chromosomes have less time to actively search for partners, and are less able to delay synapsis in response to nonhomology, as they age. However, younger *pph-4.1* mutant animals show no increase in autosomal pairing levels relative to older animals. Therefore we infer that young *pph-4.1* mutants retain the ability to delay synapsis in the absence of homologous pairing, but this delay does not lead to higher pairing levels due to the absence of PPH-4.1.

The SUN-1 protein is normally phosphorylated during the transition zone and early pachytene, and meiotic errors are correlated with persistence of SUN-1 phosphorylation [8]. We therefore tested whether the phosphorylation state of SUN-1 in *pph-4.1* mutants changed in correlation with the shortened transition zone in older germlines, using an antibody that specifically detects SUN-1 phosphorylated on Ser8 (SUN-1:Ser8p) (Figure 7). We measured the proportion of the gonad occupied by the SUN-1:Ser8p signal at 24 h, 48 h, and 72 h post-L4 as an indication of the persistence of SUN-1 phosphorylation. At all timepoints, we observed a significant increase in the proportion of the meiotic zone (from TZ entry to cellularization) containing cells positive for SUN-1:Ser8p in *pph-4.1* mutants compared to wild-type (Figure 7B). SUN-1:Ser8p is limited to nuclei with a transition zone or early pachytene appearance in all wild-type gonads, and in *pph-4.1* mutants at 24 h post-L4. However, at 72 h post-L4, *pph-4.1* mutant gonads also contain late pachytene nuclei with SUN-1:Ser8p staining (Figure S7B). This observation explains the persistence of SUN-1:Ser8p-positive nuclei in aged *pph-4.1* mutants with shorter transition zones and suggests that SUN-1 phosphorylation and nuclear morphology are uncoupled in older *pph-4.1* mutants. In contrast to the extension of SUN-1:Ser8p, nuclei positive for SUN-1:Ser12p were dramatically reduced in 72 h post-L4 *pph-4.1* gonads (Figure S7A), indicating that SUN-1 phosphorylation at Ser8 and Ser12 are independently regulated in *pph-4.1* animals. The persistence of SUN-1:Ser8p beyond its normal range suggests the possibility that PPH-4.1 is normally required for its dephosphorylation. Further work will be required to test for direct interactions between PPH-4.1 and SUN-1. We noted a significant increase in the proportion of SUN-1:Ser8p in wild-type worms at 48 h and 72 h post-L4 compared to 24 h post-L4. This observation suggests that aging presents intrinsic difficulties to meiosis, thus prolonging the time meiotic tasks take to complete. This age effect agrees with previous observations that show higher rates of apoptosis (a sign of meiotic errors) with increasing maternal age [40]. Taken together, these results imply a role for PPH-4.1 in maintaining correct meiotic progression with advancing maternal age.

## Discussion

This study has demonstrated multiple requirements for PPH-4.1 in essential aspects of meiotic prophase chromosome dynamics. In the absence of PPH-4.1 activity, autosomal pairing is reduced and promiscuous synapsis occurs between non-homologous chromosomes or within single chromosomes folded in half. Furthermore, DSB formation and crossover repair are not only defective without PPH-4.1 but deteriorate even further with advancing age. Our results explain the earlier observation of univalent chromosomes in a *C. elegans* PPH-4.1 knockdown [16] as the aggregate outcome of failures in all of these processes.

The defect in autosomal pairing in the absence of PPH-4.1 has multiple possible causes. Mutations in *plk-2* [41], *sun-1* [42], *hal-2* [43], and the SC component *htp-1* [29] have all been shown to compromise synapsis-independent pairing. Defective phosphoregulation of any of these proteins could cause defects in homologous pairing. Rad53, the budding yeast homolog of CHK-2, is dephosphorylated by PP4 to turn off the S phase checkpoint during the mitotic cell cycle [44]. It is possible that *C. elegans* CHK-2 or its substrates could have altered activity in *pph-4.1* mutants, leading to defects in homologous pairing.

Previous studies in budding yeast showed that two SC components, Hop1 and Zip1, become hyperphosphorylated in the absence of PP4 [17], [45]. Mammalian SC components HORMAD1 and HORMAD2 undergo developmentally-regulated phosphorylation [46] proposed to be part of a synapsis-monitoring system, as phosphorylated HORMAD1 is preferentially found on unsynapsed axes. Mutations in the *C. elegans* SC axial element proteins HIM-3 and HTP-1 have also been shown to cause nonhomologous synapsis of the autosomes [28]–[30]. While little functional information exists about SC phosphorylation, it is possible that dephosphorylation of SC components by PPH-4.1 plays a role in the restriction of SC assembly to homologous axes.

The number of homologous recombination sites marked by RAD-51 foci drop precipitously in *pph-4.1* and *pph-4.1; rad-54* mutant animals, indicating that normal DSB initiation depends on PPH-4.1. Interestingly, *rad-54* single mutants also showed an age-related drop in RAD-51 foci in mid-meiotic prophase. Recent studies showed that mutations in *rad-54* and other genes that cause a block in CO repair result in perdurance of the zone in which programmed DSBs are made [12], [13]. This suggests that the number of DSBs we observe in *rad-54* mutants do not reflect the wild-type level of DSBs, but rather an increased number due to a longer period of DSB initiation. We observed the same levels of RAD-51 foci up to zone 3 between young and old *rad-54* animals, whereas older animals have fewer foci in zones 4, 5, and 6. This suggests that the later prolongation of DSB formation, rather than the intrinsic mechanism of DSB initiation, specifically degrades with age. In contrast, the significantly lowered number of DSBs in *pph-4.1; rad-54* double mutants compared to *rad-54* single mutants demonstrates a strong dependence on PPH-4.1 activity for DSB initiation.

COSA-1 foci, like RAD-51 foci, are less numerous in *pph-4.1* worms compared to wild-type worms, and decrease further with maternal age. Our results strongly suggest that nonhomologous synapsis and reduced DSB formation contribute jointly and independently to the reduction of COSA-1 foci, with impaired DSB formation responsible for the age dependence. It is thought that each COSA-1 focus marks the site of a future chiasma in normal meiosis [37]. However, in *pph-4.1* mutants, the observed number of chiasmata falls short of the number predicted by COSA-1 focus numbers, in an age-dependent manner. The simplest explanation is that in *pph-4.1* mutants, COSA-1 foci do not always mature into chiasmata, with the chance of failure increasing over time. Additionally, inducing DSBs with γ-irradiation does not promote bivalent formation in excess of non-irradiated controls, despite the presence in *pph-4.1* mutants of homologously synapsed X chromosomes and some autosomes. Taken together, these lines of evidence indicate that PPH-4.1 plays a role in CO formation in addition to its role in DSB initiation. Furthermore, budding yeast PP4 has been shown to promote single-end invasions [17] and DNA synthesis steps of DSB repair [47]. These functions of PP4 may be conserved during CO formation in meiosis.

Loss of the *C. elegans* DSB-promoting factor DSB-2 [12] also produces defects in DSB and CO formation that worsen with age. DSB-2 contains several SQ motifs that are potentially substrates for the ATM/ATR DNA damage kinases. In budding yeast, Mec1 and Tel1 (ATM/ATR) phosphorylate Rec114, which limits DSB formation by Spo11 [48]. It will be interesting to see whether PPH-4.1 is required to dephosphorylate DSB-2 or its homolog DSB-1 [13] to create normal levels of DSBs. It is possible that the age effects in *dsb-2* mutants, as well as in the *rad-54* single mutation shown by the current study, are due to an increased sensitivity to as-yet unknown factors that accumulate or diminish over time. The persistent phosphorylation of SUN-1 at Ser8 raised the possibility that SUN-1:Ser8p is a substrate of PPH-4.1. SUN-1:Ser8p has been shown to be a part of the checkpoint coupling formation of CO intermediates with meiotic progression [12]. Phosphomimetic versions of SUN-1 have been shown to extend the transition zone length, similar to young *pph-4.1* mutants [8]. However, *sun-1* phosphomimetic mutants differ from *pph-4.1* mutants in that they do not show prominent defects in pairing, synapsis, or RAD-51 focus levels. The multiple, distinct meiotic defects of *pph-4.1* mutants indicate that SUN-1 is not likely to be the only substrate of PPH-4.1.

Our observation that SUN-1:Ser8p persists longer with increasing age in wild-type animals suggests an intrinsic age-related decrease of meiotic competence, which is normally accommodated through multiple checkpoint mechanisms but is unmasked in various mutant backgrounds including *pph-4.1*. The age-dependent decrease we have shown in the probability of COSA-1 foci maturing into chiasmata is interesting in light of this possibility. Since our study demonstrates a situation in which chiasma formation fails at a relatively late stage, markers of presumptive CO sites such as MLH-1 foci may outnumber chiasmata in systems where the ability to cope with meiotic errors is compromised. Although this is not likely to be the case in normal human male or female meiosis [49], [50], our results suggest the usual 1∶1 correspondence between MLH1 or COSA-1 foci and chiasmata can break down in pathological situations. The several roles of *pph-4.1* revealed in the current study are presumably attributable to hyperphosphorylation of one or more proteins required for proper meiotic prophase functions; current and future studies will identify these substrates of PPH-4.1 and illuminate how the balance of phosphorylation and dephosphorylation regulates the dynamic activities of chromosomes in meiosis.

## Materials and Methods

### *C. elegans* strains and conditions

*C. elegans* strains were grown with standard procedures [38] at 20°C. Wild-type worms were from the N2 Bristol strain. Mutations, transgenes and balancers used in this study are as follows:

**LGI**: rad-54(ok617); **LGII**: meIs8 [Ppie-1::GFP::cosa-1 + unc-119(+)], icmSi18[Ppph-4::pph-4.1(WT) + unc-119(+)], icmSi20[Ppph-4::pph-4.1(D107A) + unc-119(+)], icmSi22[Ppph-4::pph-4.1(R262L) + unc-119(+)], **LGIII**: pph-4.1(tm1598), hT2[bli-4(e937) let-?(q782) qIs48]; **LGIV**: syp-2(ok307), spo-11(me44); **LGV**: nT1[unc-?(n754) let-?(m435)]; **Unknown LG**: opIs263[Prpa-1::rpa-1::YFP + unc-119(+)].

For mutant analyses, we used homozygous mutant progeny of heterozygous parents. For all cytological assays we stringently age-matched worms by picking young adult hermaphrodites to single plates and allowing them to lay eggs for 3 hours. F1 self-progeny from this 3-hour laying period were picked from these plates at the L4 larval stage, 51–54 h after the beginning of the egg-laying period and analyzed at 24, 48, or 72 hours after the L4 stage.

### Transgenic lines

To create the transgenic *pph-4.1* constructs, we obtained synthesized DNA (GenScript) starting 311 bases upstream of the initiation ATG, and ending 591 bases downstream of the stop codon, with introns 4 and 5 removed. This fragment was cloned into plasmid pCFJ151 at the AflII restriction site. Single-site mutagenesis was performed by PfuUltra mutagenesis PCR (Stratagene) with primers containing 1- or 2-base mismatches in the relevant codons (D107A: GAC→GCT; R262L: AGA→CTA) for the creation of phosphatase-dead mutations. Single-copy insertions of transgenes into chromosome II was performed using strain EG6699 as described [51].

### Antibodies and cytology

The following antibodies used in the present study have been described previously: HIM-8 [11], HTP-3 [52], SUN-1:Ser8p and SUN-1:Ser12p [7], SYP-1 [53], ZIM-2 and ZIM-3 [24]. The RAD-51 antibody was rabbit polyclonal from SDIX/Novus Biologicals, cat# 29480002, lot# G3048-009A02, used at 1∶1000. The HIM-3 antibody, was rabbit polyclonal from SDIX/Novus, catalog # 53470002, used at 1∶500 dilution.

For all cytological preparations, we followed protocols described in [54]. Images were acquired using either a DeltaVision OMX v2 (for 3D-SIM images) or personalDV microscope (Applied Precision/GE Healthcare), using 60x or 100x oil immersion objectives (Olympus) and immersion oil (LaserLiquid, Cargille) at a refractive index of 1.513. For 3D-SIM, Z spacing was 0.125 µm; raw images were reconstructed using the softWoRx suite. For conventional widefield images, the Z spacing was 0.2 µm, and raw images were subjected to deconvolution. Image post-processing involved linear intensity scaling and maximum-intensity projection; in some color-blended maximum intensity projections (Figure 4) the DAPI channel was locally underweighted to better visualize SC staining. Whole-gonad images are montages of sequentially-acquired panels, composited using GIMP with “lighten” mode, with individual linear scaling parameters applied to each panel. For DAPI body counting, completely resolvable contiguous DAPI-positive bodies were counted in three-dimensional stacks; with this criterion, chromosomes that happen to be touching can occasionally be counted as a single DAPI body. Homologous pairing quantitation was performed as described [11]. Statistical significance of pairing was assessed by 2-tailed *t* tests comparing like zones between conditions.

### γ-irradiation and DAPI body counting

Worms at were exposed to 10 Gy (1000 rad) of γ-irradiation using a ^137^Cs source. For visualization of RAD-51 foci, worms were irradiated at 24 h post-L4, and fixed and processed for RAD-51 immunofluorescence 2 h after irradiation. For DAPI body counting, worms were irradiated at 20 h post-L4, fixed and DAPI-stained 18 h after irradiation, and imaged for scoring of DAPI bodies as above. For COSA-1 counting, worms were irradiated 18 h post-L4, and fixed and stained 8 h post-irradiation.

### COSA-1 foci and chiasmata

For the adjustment calculation, we inferred chiasmata numbers from DAPI body counts by subtracting the DAPI body count from 12. To obtain the distribution of chiasmata that would have been expected in those same nuclei from our COSA-1 counts, given a 100% probability of COSA-1 foci becoming a CO, we normalized the numbers of nuclei scored for COSA-1 to the numbers of nuclei scored for DAPI bodies. COSA-1 counts of 7 or 8 (found in 13 nuclei total out of 1360) were corrected to counts of 6. We then calculated an adjusted distribution of expected chiasma numbers for probabilities less than 100% for COSA-1 foci to mature into chiasmata. For this analysis we assume each COSA-1 site in each nucleus has an equal and independent chance (P_success_) of maturing into a chiasma. Given a starting number of nuclei *N* with *k* COSA-1 foci (*N_k_*), we calculate the adjusted number *N'_k_* for a P_success_ value of *p* from the sum of all nuclei *N_m_* multiplied by the corresponding factors derived from the binomial distribution:
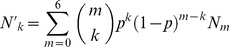



Optimal P_success_ values for the 24 h and 72 h distributions were found by minimizing the sum of squared differences between the observed DAPI body counts and the predicted counts given the value of P_success_. Adjusting for these values of P_success_ gave predicted chiasma distributions that more closely match the observed DAPI body numbers.

#### Quantitation of SUN-1 and transition zone lengths

The percent of gonads positive for crescent-shaped nuclei and for SUN-1:Ser8P staining was calculated by taking transition zone entry as a start point (or the first gonad column with a majority of nuclei positive for SUN-1:Ser8P staining, in gonads that lacked transition zone nuclei), and measuring the length to the point in the gonad where more than half the nuclei in a column are positive for crescent-shaped nuclei or SUN-1:Ser8P. This distance is divided by the entire meiotic length (from the start point to the beginning of cellularization).

## Supporting Information

Figure S1 Expression of PPH-4.1 protein and qRT-PCR analysis of pph-4.1 transcription. (A) PPH-4.1 protein was absent in homozygous mutant adult lysates derived from heterozygous parents, indicating no maternal PPH-4.1 proteins remain at detectable levels in homozygous mutant adults. All PPH-4.1 proteins carrying point mutations were expressed at wild type levels. Western blot using lysates of 30 worms prepared from the indicated strains was probed with PPH-4.1 antibodies. A cross-reacting band is indicated by an open triangle. (B) Since we observed that the meiotic defects worsen with age in pph-4.1 mutants, we considered the possibility that younger homozgous mutants still carry maternal deposits of pph-4.1 mRNA from heterozygous parents whereas older homozygous mutants run out of this pool and thus show a more severe phenotype. To examine this possibility, we performed qRT-PCR analysis. qRT-PCR 30 demonstrates only a negligible retention of pph-4.1 mRNA in pph-4.1 homozygous mutants. Younger pph-4.1 mutants did not contain more pph-4.1 mRNA than older pph-4.1 mutants. Two primer sets were used to determine the relative levels of mRNA in animals homozygous and heterozygous for pph-4.1(tm1598). Statistical significance was assessed by a one-way ANOVA. Between-sample comparisons (Tukey's multiple comparisons test) of values obtained from primer set 4 demonstrated no significant difference between 24 h and 72 h post-L4 for either homozygous or heterozygous mutants, while the difference between homozygous and heterozygous mutants at the same timepoint was significant (p<0.01). Primer set 5 gave results similar to set 4. (C) The location of qRT-PCR primers are shown superimposed on the pph-4.1 gene structure.
(EPS)

Figure S2 Pairing in pph-4.1 mutants. (A) The pairing centers of chromosomes I and IV, detected with staining against the protein ZIM-3, are often mispaired in early pachytene pph-4.1 oocytes (right) in contrast to wild-type cells (left). (B) The right end of the X chromosome, detected by FISH, also achieves high levels of pairing in pph-4.1 mutants. Bars show the mean value of the individual data points (black squares). Three gonads were scored for each genotype. The numbers of nuclei scored for zones 1, 2, 3, 4, and 5 are as follows: for wild-type, 144, 103, 208, 214, and 134; for pph-4.1, 111, 140, 123, 118, and 115. (C) The higher rate of X pairing in early prophase persists into diakinesis. 75 nuclei from pph-4.1 animals at 24 h post-L4 were scored using FISH to detect the X chromosome and chromosome V. The numbers of paired and unpaired chromosomes are shown. The frequency of X chromosome bivalency at diakinesis is significantly higher than that of chromosome V.
(EPS)

Figure S3 Synaptic configurations of wild-type and pph-4.1 mutants visualized with 3D-SIM. A, Wild-type nuclei in both early and late pachytene are fully synapsed into six pairs in all ten measured nuclei of each stage. B, pph-4.1 mutant nuclei display varying degrees of visible synaptic aberration, indicated by diagrams below each nucleus based on manual tracing. Nine out of ten nuclei in the early pachytene region, and six out of ten nuclei in the late pachytene region, show presumptive foldback synapsis (short SCs) or multivalent synaptic configuration. The top left early pachytene nucleus, and the top and bottom left late pachytene nuclei, are identical to those used in Figure 4.
(TIF)

Figure S4 HTP-1/2 and HIM-3 load normally onto chromosomes in pph-4.1 mutants. Top, immunofluorescence staining of axial element protein HTP-3 (middle) and HTP-1/2 (middle) shows complete overlapping localization (merged, right) in both wild-type and pph-4.1 mutant oocytes. Bottom, immunofluorescence of HTP-3 and HIM-3 shows equivalent patterns in both wild-type and pph-4.1 mutant oocytes.
(EPS)

Figure S5 RPA-1 localization to chromosomes is decreased in pph-4.1 mutants, in a manner similar to RAD-51 foci. Meiotic nuclei from the pachytene region are shown from rpa-1:YFP (left) and rpa-1:YFP; pph-4.1 (right) animals. Upper images shows dual staining with DAPI (magenta) and RPA-1:YFP (green); lower images show the RPA-1:YFP channel in grayscale for better visibility.
(EPS)

Figure S6 Illustration of semi-automated counting of RAD-51 foci in a rad-54 gonad at 24 h post-L4. (A) Nuclear volumes that have been automatically identified are outlined in yellow; RAD-51 foci, constrained to lie within the 3D convex hull of nuclear points, are outlined in violet circles. Examples of mis-identified nuclei requiring manual correction and counting are indicated with red outlines. DAPI staining is shown as inverse (dark staining  =  high intensity); RAD-51 foci are shown in green. Numbers on axes correspond to pixel number. (B) A subset of nuclei (inset from A) is shown with the color scheme from the main text (DAPI shown in violet; RAD-51 foci shown in green).
(EPS)

Figure S7 Meiotic progression, synapsis, and SUN-1 phosphorylation are altered in aged pph-4.1 mutants. (A) Gonads from wild-type (left) and pph-4.1 (right) at 24 h and 72 h post-L4 demonstrate the drastic loss of transition zone nuclei marked by SUN-1:Ser12P in older pph-4.1 animals. The distal end of the gonad is shown, comprised of (from left to right) the mitotic zone, the leptotene/zygotene transition zone, early pachytene, and late pachytene. Nuclei with SUN-1:Ser12P signals are demarcated with a blue dotted line. In pph-4.1 mutants at 72 h post-L4, SYP-1 immediately appears on the entire length of chromosomes after the mitotic cell cycle. In wild type gonads, SYP-1 is first detected as foci and gradually elongates into full stretches of the SC during the transition zone. At 24 h post-L4, pph-4.1 gonads more closely resemble wild-type gonads, indicating this change is age-specific. (B) Gonad regions corresponding to early pachytene in wild-type were images in each genotype. SUN-1:Ser8P staining (middle column; green in merged images at right) is limited to transition zone (clustered nuclear morphology with no individual chromosome distinguishable) or early pachytene (clustered nuclear morphology with a few chromosomes distinguishable) nuclei in pph-4.1 at 24 h post-L4 or in wild-type gonads at both 24 h and 72 h post-L4. In contrast, SUN-1:Ser8P staining surrounds nuclei with late-pachytene (evenly distributed, individual chromosomes) appearance in pph-4.1 oocytes at 72 h post-L4.
(EPS)

Table S1 Progeny viability, percentage of male progeny, larval arrest, and Dumpy (Dpy) progeny in pph-4.1 mutants compared to wild-type control. For each category, the percentage of worms with the given phenotype is shown followed by the number of worms scored in parentheses. Embryonic inviability is derived from autosomal missegregation at meiosis as well as mitotic defects. PPH-4.1 is essential for centriole functions during male spermatogenesis and embryogenesis [16], and thus embryonic inviability of pph-4.1 mutant is likely due to the combined effect of meiotic and mitotic defects. Male (XO) or Dpy (XXX) self-progeny indicates X chromosome missegregation, whereas progeny arrested at larval stage is likely to indicate autosomal aneuploidy or other mitotic defects. Crossprogeny of mutant hermaphrodites with wild-type males had a modest but significant rescue of embryonic lethality (two-tailed chi-square test, P<0.0001).
(PDF)

Movie S1 The X chromosome synapses homologously in pph-4.1 mutants. The movie shows a series of Z sections at 0.2 µm spacing taken with conventional deconvolution fluorescence microscopy of a pph-4.1 mutant gonad at late pachytene. HTP-3 is shown in red; SYP-1 is shown in green; HIM-8 staining marking the pairing center end of the X chromosome is shown in blue. The X chromosome pairing center appears as a single paired spot at or near the end of a continuous stretch of SC.
(MOV)

Text S1 Supplemental experimental procedures, including protocols for Western Blotting, qRT-PCR, FISH, RPA-1:YFP imaging, and RAD-51 focus quantitation.
(PDF)
