# Supplementary figures and images for: Protein Phosphatase 4 Promotes Chromosome Pairing and Synapsis, and Contributes to Maintaining Crossover Competence with Increasing Age
Source: PLoS Genet. 2014 Oct 23;10(10):e1004638. doi: 10.1371/journal.pgen.1004638 (PMC4207613; doi:10.1371/journal.pgen.1004638)

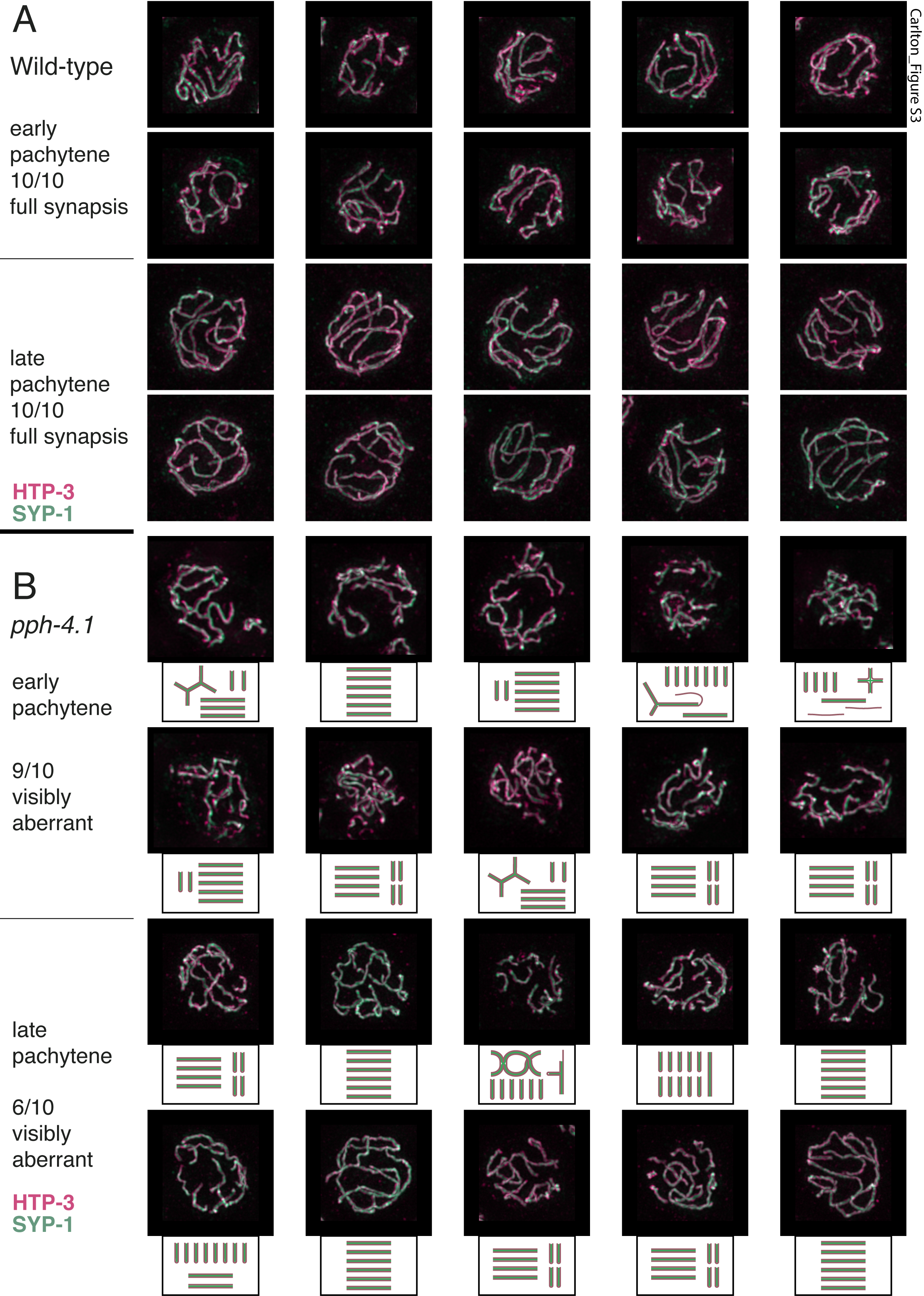

Supplement: Figure S3 — Synaptic configurations of wild-type and pph-4.1 mutants visualized with 3D-SIM. A, Wild-type nuclei in both early and late pachytene are fully synapsed into six pairs in all ten measured nuclei of each stage. B, pph-4.1 mutant nuclei display varying degrees of visible synaptic aberration, indicated by diagrams below each nucleus based on manual tracing. Nine out of ten nuclei in the early pachytene region, and six out of ten nuclei in the late pachytene region, show presumptive foldback synapsis (short SCs) or multivalent synaptic configuration. The top left early pachytene nucleus, and the top and bottom left late pachytene nuclei, are identical to those used in Figure 4. (TIF) [file pgen.1004638.s003.tif]
