# Supplementary material for: Protein Phosphatase 4 Promotes Chromosome Pairing and Synapsis, and Contributes to Maintaining Crossover Competence with Increasing Age
Source: PLoS Genet. 2014 Oct 23;10(10):e1004638. doi: 10.1371/journal.pgen.1004638 (PMC4207613; doi:10.1371/journal.pgen.1004638)
Supplement: Table S1 — Progeny viability, percentage of male progeny, larval arrest, and Dumpy (Dpy) progeny in pph-4.1 mutants compared to wild-type control. For each category, the percentage of worms with the given phenotype is shown followed by the number of worms scored in parentheses. Embryonic inviability is derived from autosomal missegregation at meiosis as well as mitotic defects. PPH-4.1 is essential for centriole functions during male spermatogenesis and embryogenesis [16], and thus embryonic inviability of pph-4.1 mutant is likely due to the combined effect of meiotic and mitotic defects. Male (XO) or Dpy (XXX) self-progeny indicates X chromosome missegregation, whereas progeny arrested at larval stage is likely to indicate autosomal aneuploidy or other mitotic defects. Crossprogeny of mutant hermaphrodites with wild-type males had a modest but significant rescue of embryonic lethality (two-tailed chi-square test, P<0.0001). (PDF) [file pgen.1004638.s008.pdf]

Table S1

***pph-4.1* visible phenotypes**

Genotype	%Embryonic viability	%Male progeny	%Larval arrest progeny	%Dpy progeny
wild-type (N2) (self-progeny)	100 (1244)	0.16 (1244)	0 (1244)	0 (1244)
pph-4.1(tm1598) (self-progeny)	3.0 (1374)	23.8 (42)	6.7 (42)	2.4 (42)
pph-4.1(tm1598) (mated to wild-type males)	9.8 (1065)	63.7 (104)	0 (104)	0 (104)
