# Supplementary material for: Protein Phosphatase 4 Promotes Chromosome Pairing and Synapsis, and Contributes to Maintaining Crossover Competence with Increasing Age
Source: PLoS Genet. 2014 Oct 23;10(10):e1004638. doi: 10.1371/journal.pgen.1004638 (PMC4207613; doi:10.1371/journal.pgen.1004638)
Supplement: Text S1 — Supplemental experimental procedures, including protocols for Western Blotting, qRT-PCR, FISH, RPA-1:YFP imaging, and RAD-51 focus quantitation. (PDF) [file pgen.1004638.s010.pdf]

Text S1

Supplemental Experimental Procedures

Western blotting:

For each lane, 30 worms were picked into M9 buffer with 0.01% Tween-20, washed three times in the same buffer and then resuspended in SDS-PAGE sample buffer. SDS-PAGE was performed using a 10% Supersep gel (Wako). Proteins were transferred to PVDF membrane, and the membrane was blocked with 0.2% BSA in PBST, probed with PPH-4.1 primary antibodies and detected with HRP-conjugated secondary antibody, diluted in Can Get Signal (Toyobo) immunoenhancer solution A and B, respectively. HRP signals were detected by ECL select (GE Healthcare). The α-PPH-4.1 polyclonal antibody was generated by injecting rabbits with PPH-4.1's N-terminal 16 AAs. The PPH-4.1 antibody was used at 1:100 dilution for western blotting.

qRT-PCR: pph-4.1 worms and their heterozygous mutant siblings were separated onto different plates at the L4 larval stage and grown for either 24h or 72h. Animals were collected and washed 3 times in M9, then total RNA was extracted from approximately 150 animals. RNA was extracted using the TRI reagent (Sigma) and cDNA synthesized using the SuperScript III Reverse Transcriptase (Invitrogen). Fast SYBR Green Real Time Quantitative PCR was carried out using the StepOnePlus Real-Time PCR System (Applied Biosystems) and analyzed using the Standard Curve method. *tba-1*(α-tubulin) and *pph-4.1(tm1598)* 24h post L4 homozygous mutant *pph-4.1* mRNA levels were used for normalization. Primers used were as follows: “Set 4”: Forward: AACAC-CAACTATTTGTTCCTCGG, Reverse: AGTGCGAGAAGTAGAAGGAATGTC spanning exons 3 and 4. “Set 5”: Forward: GCTCGTGAAATCCTAGCGGAAGA, Reverse: AGAACTGGCCGTG-GATGT spanning exons 2 and 3.

X chromosome right end FISH:

FISH probes recognizing the right end of the X chromosome, used in Figure S2B and S2C were generated as previously described [3] using cosmids T23E7, F20B4, F15G10, and K09G11 obtained from the Sanger Center.

RPA-1:YFP fluorescence imaging: Worms were dissected and frozen as previously described [54]. Slides were fixed in cold methanol for 1 min after freeze-crack and further fixed in 4% formaldehyde for 15 min, followed by DAPI staining for 5 min. Slides were then washed in PBST for 10 min and mounted for observation.

Quantitation of RAD-51 focus number:

RAD-51 counting: For early zones (1 and 2) with very few RAD-51 foci, manual counting was performed. For zones 3 and above, custom programs written in GNU Octave were used to semi-automatically count the number of RAD-51 foci in each nucleus. Code is available upon request and will be placed at github.com/pmcarlton. The programs proceed via the following steps: (1) nuclear centers are found by identifying all voxels above a threshold (calculated with the Otsu method [55] on a maximum-intensity projection image) that are also local maxima; these pixels are then subjected to a gravitational attraction routine that collapses clouds of pixels into small clusters that with few exceptions lie at the center of imaged nuclei. (2) The original positions of all the pixels that contributed to a cluster that fall within a certain radius of the center are used to define a convex hull that represents the nuclear volume. (3) Positions of all RAD-51 foci are calculated by thresholding as in step 1. (4) RAD-51 foci are assigned to the convex hull in which they are enclosed. Detected foci not located inside any convex hulls are rejected as background spots. The convex hull outlines and number of foci per nucleus are displayed as 2D projections for each image data file, and used during visual inspection of the 3D data to correct or reject mistaken counts.

Supporting References

55. Otsu N (1975) A threshold selection method from gray-level histograms. Automatica 11: 23–27.
